# Thought Control Ability Moderates the Effect of Mind Wandering on Positive Affect via the Frontoparietal Control Network

**DOI:** 10.3389/fpsyg.2018.02791

**Published:** 2019-01-25

**Authors:** Hong He, Qunlin Chen, Dongtao Wei, Liang Shi, Jiang Qiu

**Affiliations:** ^1^Key Laboratory of Cognition and Personality (Ministry of Education), Chongqing, China; ^2^School of Psychology, Southwest University, Chongqing, China

**Keywords:** mind wandering, thought control ability, positive affect, frontoparietal control network, moderating effect

## Abstract

Mind wandering is a phenomenon that involves thoughts shifting away from a primary task to the process of dealing with other personal goals. A large number of studies have found that mind wandering can predict negative emotions, but researchers have seldom focused on the positive role of mind wandering. The current study aimed to explore the relationships among mind wandering, emotions and thought control ability, which is the ability to inhibit one’s own unpleasant or unwanted intrusive thoughts. Here, we collected resting-state functional magnetic resonance imaging (rsfMRI) data from 368 participants who completed a set of questionnaires involving mind wandering, thought control ability and positive or negative emotions. The results revealed that (1) rsfMRI connectivity features related to thought control ability and mind wandering could divide individuals into two groups: HMW (high mind-wandering) group and LMW (low mind-wandering) group. The HMW group scored lower in thought control ability (TCA), higher in negative emotion (NE) and lower in positive emotion (PE) than the LMW group. (2) TCA moderated the association between MW and positive affect (PA). (3) Two groups exhibited different segregation within key nodes (SWKN) of the frontoparietal control network (FPCN), and the subsequent analysis showed that the SWKN of the FPCN was negatively correlated with PA. These findings indicate that TCA moderates the effect of mind wandering on affect via the FPCN, which may have important implications for our understanding of the positive role of mind wandering.

## Introduction

Mind wandering refers to a shift in thoughts away from the current task to the process of dealing with internal activities or goals regarding places, people or other situations that are not limited to the present moment (Smallwood and Schooler, 2006; Smallwood et al., 2007; Killingsworth and Gilbert, 2010). This experience exists in every aspect of our lives, and researchers have suggested that humans spend almost half of their waking hours mind wandering (Klinger and Cox, 1987; Klinger, 1999; Killingsworth and Gilbert, 2010; Singer et al., 2014). Generally, mind wandering is viewed as having a harmful role in impeding the performance of tasks requiring executive attention, as well as causing an increased risk of affective dysfunction (Smallwood et al., 2003; Killingsworth and Gilbert, 2010). However, other studies have shown that mind wandering provides some benefits to human behavioral performance, such as creative thinking (Baird et al., 2012), problem-solving (Stawarczyk et al., 2011a; Ruby et al., 2013b) and avoiding pain (Kucyi et al., 2013). Mind wandering can be a double-edged sword for accomplishing tasks and for emotion. In the current study, we aimed to explore whether mind wandering has a dual influence on affect and whether this double-sided effect can be affected by thought control ability, and reflects at the individual differences in brain functional connectivity patterns.

Previous studies have consistently connected mind wandering to negative affect (Watts et al., 1988; Smallwood et al., 2007, 2009; Killingsworth and Gilbert, 2010; Smallwood and O’Connor, 2011; Stawarczyk et al., 2011a). The occurrence of mind wandering has been found to increase the extent to which subjects rate their negative emotions (Killingsworth and Gilbert, 2010). Prior sadness leads to incidents of mind wandering (Poerio et al., 2013), and the induction of negative affect predicts the frequency of mind-wandering episodes (Stawarczyk et al., 2013). The temporal focus of off-task thought is also related to mood. The content of mind-wandering often shifts from a focus on the future to one on the past when the mood is low (Smallwood and O’Connor, 2011). In contrast, some researchers have indicated that mind wandering can both alleviate negative emotions and promote positive emotions. Smallwood et al. found that positive states also trigger mind wandering apart from negative states (Smallwood et al., 2009; Smallwood and O’Connor, 2011). Positive emotions make it easy to remember positive past experiences, and memory extraction reinforces people’s current moods (Schacter, 1996). In turn, frequent positive mind wandering could also increase positive emotions (Cohn et al., 2009). Researchers (Ruby et al., 2013a; Engert et al., 2014) have shown that when off-task thought is focused on the future, then it tends to be linked to more positive aspects. Researchers were generally concerned about the relationship between a single emotion scale and a single mind wandering scale. However, mind wandering and emotions contain multiple traits. We studied this by assessing a broad array of emotion and mind-wandering scales, and then confirmatory factor analysis (CFA) was conducted to create a few factors as previous study did (Morelli et al., 2017).

In summary, since there have been different opinions on the relationships between mind wandering and emotions, we wondered whether the relationship between mind wandering and affect is affected by thought control ability, which is the ability of people to control their own unpleasant or unwanted intrusive thoughts (Luciano et al., 2005; Williams et al., 2010). The relationship between thought control ability and emotion is contrary to the relationship between mind wandering and emotion. Subjects who report they have higher thought control ability have lower anxiety and less negative affect (Luciano and Algarabel, 2006; Gootjes and Rassin, 2014), and this relationship is shown to be significantly correlated with several dimensions of negative general psychological symptoms, such as anxiety and depression (Luciano et al., 2005; Peterson et al., 2009). In addition, increased thought control ability is associated with higher scores in optimism and social connectedness, and they also have a more positive affect (Gootjes and Rassin, 2014). Based on the executive failure hypothesis that mind wandering occurs when the executive control system fails to fight interfering thoughts or distracts internal thoughts (Mcvay and Kane, 2009, 2010, 2012; Pachai et al., 2016; Shulley and Shake, 2016), we generated a hypothesis that thought control ability may contribute to influencing the relationship between mind wandering and emotions, as the executive deficit hypothesis suggests that differences in executive control abilities contribute to thought control ability, and individuals control the influence of unwanted thoughts by participating in executive control mechanisms that target declarative memory-related neural regions (Levy and Anderson, 2008).

Mind wandering has been found to be associated with not only activation of default mode network (DMN) but also FPCN (Christoff et al., 2009; Fox et al., 2015). Moreover, the connectivity between the FPCN and the DMN is significantly correlated with mind wandering questionnaire scores (Godwin et al., 2017). These findings reveal that mind wandering entails recruitment of both the executive system and the task-negative network of the brain. The FPCN is a network composed of executive control regions (Vincent et al., 2008), and it has been previously described as a task-positive or executive control network (Seeley et al., 2007; Corbetta et al., 2008; Vincent et al., 2008; Niendam et al., 2012; Raichle, 2015; Mittner et al., 2016). Several studies have found that the regions of FPCN are activated during mind-wandering tasks (Christoff et al., 2009; Dumontheil et al., 2010; Stawarczyk et al., 2011b). The study indicating that use transcranial direct-current stimulation (tDCS) to stimulate the regions of FPCN increases the amount of self-reported mind-wandering also supports the important role of FPCN to mind wandering (Axelrod et al., 2015). The FPCN is clearly one of the important aspects of mind wandering research. At the same time, high activation of the dorsolateral prefrontal cortex (DLPFC), which belongs to the task-related network, predicts enhanced thought control ability (Anderson et al., 2004). Given the important role of FPCN in mind wandering and thought control ability, as well as the uncertain brain mechanisms of mind wandering and emotions, we believe that the role of FPCN in the whole brain may play an important role in mind wandering and emotions. In addition, the neural mechanism of mind wandering, as one kind of spontaneous thought, may have some relationships with networks, which include the DMN, dorsal attention network (DAN), salience network (SN), ventral attention network (VAN), FPCN, and cingulo-opercular control network (COCN) (Christoff et al., 2016). As the content regulation hypothesis (Smallwood and Andrewshanna, 2013; Smallwood and Schooler, 2015) and executive failure hypothesis (Mcvay and Kane, 2009, 2010) both emphasize the important role of executive control, we aimed to use these large-scale brain networks to determine the relationships among thought control ability, mind wandering, emotions and FPCN.

There have been studies that used resting-state fMRI to examine trait mind-wandering. Mooneyham et al. (2017) supported the recruitment of default and executive networks by using a dynamic functional connectivity approach. The DMN, FPCN and limbic networks reflect the intentionality of mind wandering (Golchert et al., 2017). Smallwood et al. used seed-based functional connectivity of resting state fMRI found that the posterior core of the DMN is a hub contributing to the content of mind wandering and also found patterns of connectivity linked to the emotional tone of spontaneous thought (Jonathan et al., 2016). Increased connectivity between FPCN and DMN and increased DMN connectivity are both positively correlated with trait mind wandering (Godwin et al., 2017). As global connectivity which involves both within and outside connections of the prefrontal cortex (PFC, component of FPCN) predicts cognitive control (Cole et al., 2012), in the current study, we used a similar method - SWKN, which considers both within and between indicators of behavior related biomarkers, to explore the overall relationships among the interested networks in whole brain rather than the relationships between single networks. So far, most studies of characterizing degree of mind wandering have begun by identifying clusters of mind-wandering scales, and by then exploring the neurophysiological correlates. Still, mind wandering is a continuous behavioral data and there is no good criteria for classifying subjects. As defining subtypes of the behavioral indicators by using resting-state connectivity biomarkers has been proved to be advisable (Anderson et al., 2010; Anderson and Cohen, 2013; Drysdale et al., 2017; Wen et al., 2017), to better explore the brain patterns of individuals with different level of mind wandering and figure out their differences, we chose to cluster individuals into groups by connectivity features (CF).

In the present study, 422 participants underwent fMRI scans and out-of-scanner psychological tests, including mind-wandering-related questionnaires, the thought control ability questionnaire (TCAQ), and scales which related to negative and positive emotions. First, recent research has noted that resting-state functional connectivity can be used as a biomarker in the evaluation or prediction of individual cognitive ability (Finn et al., 2015; Drysdale et al., 2017; Shen et al., 2017). Therefore, thought control ability and mind wandering questionnaires were used to select the CF related to thought control ability and mind wandering, and then whether the functional connectivity biomarkers could classify people who had different degrees of positive or negative emotion was explored. Second, the purpose of this article was to find whether TCA measured by TCAQ could influence the relationships between mind wandering and emotions from the perspective of individual differences. Finally, we explored whether the FPCN contributes to the relationships between mind wandering and emotions. In summary, the hypothesis was that the TCA would influence the relationships between mind wandering and emotions via the FPCN. This study will deepen our understanding of the emotional role of mind wandering.

## Materials and Methods

### Participants

The participants were healthy, right-handed college students derived from an ongoing project investigating the associations among genes, brain, and behavior (GBB project) at the Southwest University (Liu et al., 2017). There were 422 native Chinese-speaking individuals, ranging in age from 16 to 26 years (119 men, mean age = 19.24 years), who completed all of our questionnaires, which were related to mind wandering, thought control ability, positive emotion and negative emotion. The participants were mostly recruited through advertisements on billboards and leaflets or through online campus on advertising. Forty-four participants were excluded because they exhibited head motion that was >2 mm maximum displacement in translation or in rotation. Consequently, a total of 368 participants (103 men, mean age = 19.38 ± 1.35) were included for analysis. This project was approved by the Institutional Review Board of the Southwest University Brain Imaging Center. Written informed consent was received from each subject (for adolescents under 18 years of age, we also obtained written informed consent and assent from their guardians), and no participant had a history of neurological disorders or psychiatric diseases, and they obtained a reward depending on the time they spent and the tasks they completed.

### Behavioral Assessment

The participants were asked to complete a series of questionnaires and psychological cognitive tasks out of the MRI scanner. The participants in the current study were 422 students who had finished all of this following scales: the Daydreaming Frequency Scale (DDFS) (Singer and Antrobus, 1972), Mind Wandering Frequency Scale (MWFS) (Wang, 2011), Positive Affect and Negative Affect Scale (PANAS) which consists of two dimensions of PA and negative affect (NA) (Watson et al., 1988), Beck Depression Inventory (BDI) (Beck et al., 1996), Beck Anxiety Inventory (BAI) (Beck et al., 1988), Urban Happiness Index Scale (UHIS) (Liu et al., 2012), Oxford Happiness Inventory (OHI) (Hills and Argyle, 2002) and the Center for Epidemiological Studies-Depression Scale (CES-D) (Radloff, 1977). The descriptive statistics for all measures are presented in Supplementary Table 1.

The thought control ability questionnaire is a 25-item questionnaire. Participants responded to statements using a 5-point Likert scale ranging from one to five. A higher score of the TCAQ means higher perceptions of participants’ about their ability to control their own thoughts rather than actual ability to control their own thoughts. The TCAQ is a one-factor inventory with a internal consistency (Cronbach’s alpha) of 0.92 and a reliability coefficient of 0.88 (Luciano et al., 2005).

### fMRI Data Acquisition

Functional MRI images were collected using a Siemens 3T Trio scanner (Siemens Medical Systems, Erlangen, Germany) with a 12-channel head coil. The 242 whole-brain resting-state functional images were obtained with the following parameters: TR/TE = 2000 ms/30 ms, FA = 90°, FOV = 220 mm × 220 mm slices = 32, thickness = 3 mm, slice gap = 1 mm, acquisition voxel size = 3.4 mm × 3.4mm × 3.4 mm. During resting-state scanning, the participants were instructed to lie down with their eyes closed and to remain awake without thinking of anything special. High-resolution 3D T1-weighted structural images were obtained using a Magnetization Prepared Rapid Acquisition Gradient-echo (MPRAGE) sequence: TR/TE = 1900 ms/2.52 ms, FOV = 256 mm × 256 mm, FA = 9°, slices = 176, thickness = 1 mm, and voxel size = 1 mm × 1 mm × 1 mm.

### MRI Data Processing

Data preprocessing was carried out by using Data Processing Assistant for Resting-State fMRI (DPARSF)^1^ (Chao-Gan and Yu-Feng, 2010) which is based on SPM8^2^. The first 10 scans were discarded to avoid modulation effects, and the remaining 232 volumes were adopted for the following analysis. Then slice timing and head motion correction were used to account for slice order effects and head movement artifacts, respectively. Furthermore, each participant’s functional images were spatially normalized to the standard Montreal Neurological Institute (MNI) EPI template space, and we regressed out the signal of white matter, the cerebrospinal fluid signal and the Friston 24-parameter (Friston et al., 1996). The data were then spatially smoothed with a 6-mm full-width at half maximum (FWHM) Gaussian kernel. Finally, the smoothed images were filtered using a typical band pass set to 0.01–0.1 Hz.

### Features Selection

The Power 264 parcellation was used, which is a template based on task-related fMRI studies (Power et al., 2011), to build spheres with 5-mm diameters centered in a series of 264 MNI coordinates as regions-of-interest (ROIs). For each ROI, one average time series was obtained by extracting from all the composed voxels. We controlled for scan coverage by excluding ROIs if the signal-to-noise ratio (SNR) was more than two standard deviations among the 264 ROIs of the subjects (12 ROIs discarded). Pearson’s ρ correlation was computed among the ROIs to generate a 252 × 252 matrix of correlation coefficients, containing 31626 unique functional connectivity values. The functional connectivity matrix is shown through a heat map after using the Fisher transformation to normalize each correlation coefficient (*z* = 0.5 ln [(1 + *r*)/(1–*r*)]) (Grady et al., 2016). The resulting matrix is a significant relatedness graph, which is a Fisher z-transformed r-matrix. We thought that a subset of 31626 functional connectivity that was significantly correlated with mind wandering and thought control ability scores most advantageously characterized the biologically meaningful mind wandering and thought control ability subtypes, some information for further analysis will be lost by using strict threshold, so a threshold of 0.001 was chosen and no correction was made. Therefore, the CF for clustering was functional connectivity, which were significantly correlated with any one of the MWFS score, the DDFS score or the TCAQ score (*p* < 0.001). And there exists the same method for defining depression subtypes (Drysdale et al., 2017). To ensure the validity of the selection method, *p* value thresholds set as 0.005 and 0.01 were considered separately and these results are presented in the Supplementary Materials.

### Statistical Analysis

For each questionnaire, select items were coded on the basis of scoring guides. For each measure, all items were added up to create a composite score. To better explore the relationships among mind wandering, thought control ability, positive emotion and negative emotion and to determine whether the selected CF could distinguish participants who had different types of personality traits, CFA was used on all composite scores for mind wandering, positive emotions and negative emotions on the 368 participants for data reduction (Supplementary Figure 1, as there have been at least two item scores for each dimension of a confirmatory factor analysis, TCAQ score itself formed a separate dimension as TCA, and we didn’t put the score into CFA). For the CFA solution, the model fit was evaluated, and the CFA results suggested the model was acceptable (Hu and Bentler, 1999). Specifically, the Tucker-Lewis Index was 0.92, the root mean-square error of approximation was 0.09, and the standardized root mean-square residual is 0.07. For the CFA solution, each absolute value of factor loading was more than 0.4, and the model fit parameter was acceptable. In addition, we multiplied each factor (e.g., PA) by its factor load, and then averaged the items for that factor (e.g., positive emotion) as Morelli et al. (2017).

To more deeply explore this structure, correlation analysis was used to measure if these three factors correlated with each other. As for moderator analysis, the lm function in the stats package (a built-in package of R) for R was employed to investigate whether there existed moderating effects of mind wandering and thought control ability on emotions^3^. The moderation models were used to test whether TCA moderated the relation of mind wandering and emotions: (1) MW as an independent variable, PE as a dependent variable, TCA as a moderator; (2) MW as an independent variable, NE as a dependent variable, TCA as a moderator; (3) MW as an independent variable, NA as a dependent variable, TCA as a moderator; (4) MW as an independent variable, PA as a dependent variable, TCA as a moderator. We used a bootstrap procedure to more accurately estimate the indirect effects (Shrout and Bolger, 2002). We created 1000 bootstrap samples, and the moderating effect was significant when confidence interval (CI) doesn’t include zero.

We were concerned about the different relationship among MW, TCA and emotions, and the different moderating effect between the two groups of people. In this study, we used the K-means clustering algorithm to cluster participants based on the selected CF, and then the Calinski-Harabasz criterion value was used to evaluate the optimal number of groups at the 0.001 threshold (Caliński and Harabasz, 1974), as well as the other two thresholds (*p* value thresholds set as 0.005 and 0.01 separately), and the cluster number was set at 1–20 by using MATLAB’s evalclusters function to find the optimal cluster^4^. The group solution of dividing individuals into groups was optimal for defining subgroups, which maximized the differences from each other. Moreover, comparing the questionnaire data differences between the groups was performed after receiving the optimal group number.

To specify the brain mechanisms of mind wandering, the DMN, FPCN, COCN, DAN, VAN, and SN were adopted, which are brain networks related to spontaneous thought (Christoff et al., 2016), as masks to explore the relationships between CF and personality traits. We chose the CF belonging to the six networks whose mean functional connectivity was positive among 368 participants to predict the classification accuracy of whole CF, this procedure proved the method of concentrating on the six network was effective. In addition, linear discriminant analysis (LDA) was used to ensure that selecting the six networks was representative in all features to explore the brain mechanisms of mind wandering. By randomly sampling nine-tenths of the individuals’ connection matrix in six networks whose constituent nodes belong to CF as training data and the remaining individuals’ data as test data, the lda function in the MASS package for R^5^ and the predict function in the stats package for R was used to compute the accuracy rate of this connection matrix predicting the population category, which was previously classified by CF in the entire brain. A ten-fold cross-validation method within sample was performed (repeated 30 times). Furthermore, we tested whether this connection matrix could separate people with similar behavioral characteristics (Supplementary Figure 3). As for the segregation index, a measure of the system segregation was calculated using the system segregation = (w–b)/w (Chan et al., 2014). However, in the current study, w is the mean value of Fisher z-transformed r between ROIs that belong to CF within the same network, and b is the mean value of Fisher z-transformed r between ROIs that belong to CF within one network to all ROIs that belong to CF in other networks. The segregation index in this study was called SWKN. Owing to the present mixed meaning of negative correlations (Schölvinck et al., 2010; Chai et al., 2012; Chan et al., 2014), and the SWKN index that we used is a method similar to system segregation index (a graph-theoretic framework), system segregation is an index to summarize values of within-system correlations in relation to between-system correlations which only retains the weight of all positive edges (Chan et al., 2014), the negative z-values were excluded from w and b, and we only chose differences of positive functional connectivity for SWKN analysis.

## Results

### Behavioral Results

The factor analysis suggested three main components: MW, PE, and NE. MW served as the individuals’ tendency to engage in mind wandering. The PE value represented the tendency of the participants to experience positive emotions. The NE value stood for the tendency of participants to experience negative emotions. The scores of each component were merged by multiplying each factor by its factor load and averaging the multiplied scores belonging to one factor. Correlation analysis showed that MW negative correlations with TCA (*r* = -0.606, *p* < 0.01), and significantly positive correlations with NE (*r* = 0.569, *p* < 0.01) as well as negatively related to PE (*r* = -0.450, *p* < 0.01). Furthermore, there was a completely opposite relationship between TCA and PE (*r* = 0.559, *p* < 0.01) as well as between TCA and NE (*r* = -0.577, *p* < 0.01), which were consistent with the findings of the previous reported (Kane et al., 2007; Killingsworth and Gilbert, 2010; Gootjes and Rassin, 2014).

### Group-Level Differences in Behavior and Functional Connectivity

**FIGURE 1:**
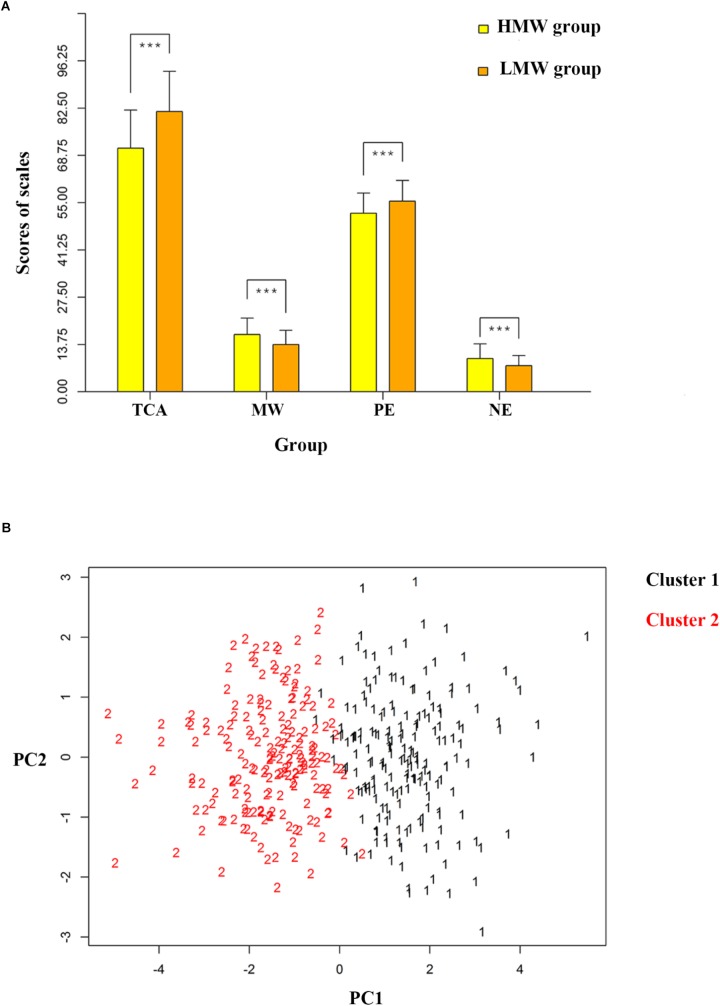
Comparison of behavior differences between two groups. **(A)** The different groups of participants divided with 0.001 significance threshold CF of each component (as indexed by the CFA solution) that were considered higher vs. lower in MW. **(B)** Scatterplot for two clusters of participants along dimensions of mind wandering- and thought control ability-related functional connectivity. MW = mind wandering, TCA = thought control ability, PE = positive emotion, NE = negative emotion, CF = connectivity feature, HMW = high mind-wandering, LMW = low mind-wandering, PC = principle component. ^∗∗∗^*p* ≤ 0.001.

**FIGURE 2:**
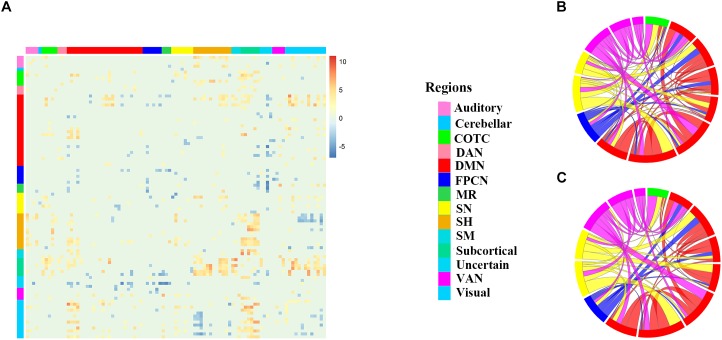
The different connectivity patterns between the two groups. **(A)** 95 × 95 *t*-test matrix of the two groups with positive functional connectivity under the 0.001 threshold (FDR corrected). Each colored square represents the significant t value of one functional connectivity between two groups. The *X*-axis and *Y*-axis represents 95 nodes, respectively, warm colors in the matrix represent increased functional connectivity and cool colors decreased functional connectivity in HMW group as compared to LMW group. **(B,C)** Chordal graphs of the connectivity in specific edges of the six networks of interest. The community membership of nodes and the loadings of functional connectivity was indicated by inner arcs and thickness of the chords, respectively. Network affiliation abbreviations: HMW = high mind wandering, LMW = low mind wandering, Auditory = Auditory network, Cerebellar = cerebellar network, COTC = cingulo-opercular task control network, DAN = dorsal attention network, DMN = default mode network, FPCN = frontoparietal control network, MR = memory retrieval network, SN = salience network, SH = somatomotor hand network, SM = somatomotor mouth network, VAN = ventral attention network. Subcortical = subcortical network, Uncertain = uncertain network, and Visual = visual network.

Cluster analysis showed that two classes was optimal by using Calinski-Harabasz criterion values (Caliński and Harabasz, 1974), the larger the Calinski-Harabasz value, the better the result, thus individuals were divided into two groups, and the Calinski-Harabasz values with cluster numbers between 1 and 20 were shown in the Supplementary Figure 5. Independent-samples *t*-test showed that the MW scores were significantly lower in one group (*N* = 180, we called it LMW for convenience of description) than the other (*N* = 188, we called it HMW for convenience of description). Scatterplot for two clusters of participants was showed in the Figure 1B. Two-tailed independent-samples *t*-tests were used to explore the difference in the brain maps between the two groups, and a significant difference was revealed among 14 networks. The statistical maps were thresholded using a false discovery rate (FDR) of *q* = 0.05 (Figure 2A), and the CF were edges involving 95 different nodes. Two other thresholds were set to demonstrate the stability of these biomarkers (see Supplementary Figure 2, the CF were edges involving 216 and 242 nodes, respectively). We also used two-tailed independent-samples *t*-tests to explore the behavioral characteristics between the two groups. Individuals in the LMW group had higher score in TCA (*t* = 6.321, *p* < 0.001), lower score in MW (*t* = -5.690, *p* < 0.001), higher score in PE (*t* = 8.960, *p* < 0.001) and lower score in NE (*t* = -5.447, *p* < 0.001) when compared with HMW group (Figure 1A). Similar results were obtained under the other two thresholds for the CF selection (Supplementary Figure 4).

Based on the networks of interest including COTC, DMN, FPCN, SN, DAN and VAN, the CF in the five networks including 10 functional connectivity could also divide people into two groups with the mean prediction accuracy of 0.64 by using ten-fold cross-validation (there was no CF consisted of DAN, we only presented five networks), and it could separate two subgroups with different degree of MW, TCA and NE as the CF in the whole brain did, but not PE. The *t*-test histogram is shown in Supplementary Figure 3. To describe the differences in the five networks of interest, the chordal graph was used to display the connection mode of the 14 ROIs, which belonged to five networks’ positive CF (Figures 2B,C), and the CF are shown in the Supplementary Figure 3. To describe the differences in the six networks of interest, the chordal graph was used to display the connection mode of the 14 ROIs. The community membership of nodes was represented by inner arcs, and the loadings of functional connectivity was indicated by the thickness of the chords. There existed different connection patterns between two groups.

### Thought Control Ability Moderates the Influence of Mind Wandering on PA

**FIGURE 3:**
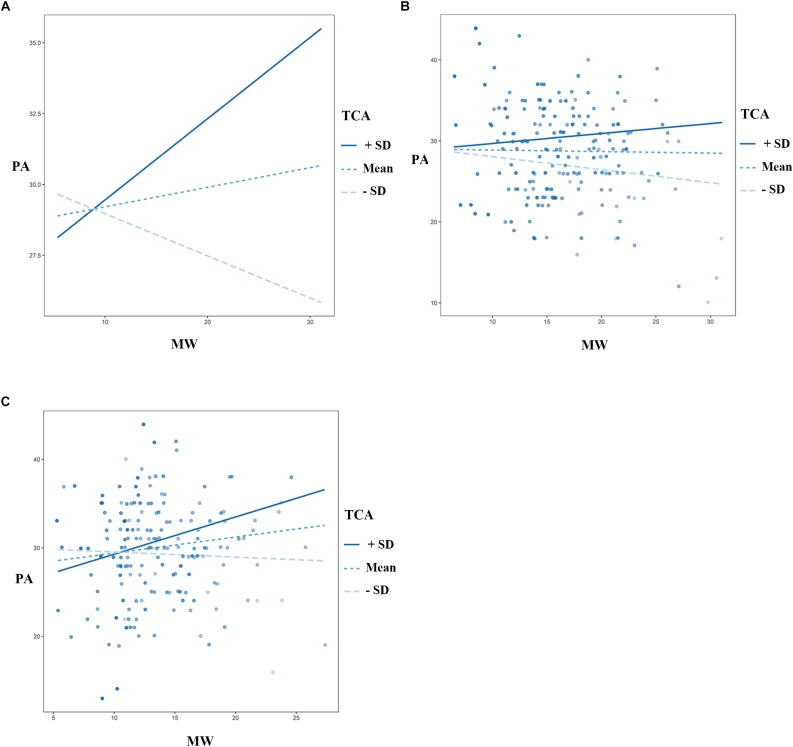
TCA moderates the effect of MW on PA (one dimension of the PANAS). **(A)** The moderating effect in all subjects. **(B)** There did not exist moderating effect in the HMW group. **(C)** The moderating effect in the LMW group. TCA = thought control ability, MW = mind wandering, PA = positive affect, PANAS = Positive Affect and Negative Affect Scale, HMW = high mind-wandering, LMW = low mind-wandering.

The results suggested a significant interaction between TCA and MW in terms of PA [β = 0.02, *SE* = 0.00, *F* = 19.98, *R*^2^ = 0.05, *p* < 0.001, 95% CI = (0.085 0.489)]. An interaction between TCA and MW also existed in the LMW group [β = 0.02, *SE* = 0.01, *F* = 7.02, *R*^2^ = 0.04, *p* < 0.01, 95% CI = (0.077 0.766)], but not the HMW group, meaning that TCA moderated the effects of MW on PA in the LMW group but not the HMW group (Figure 3), moderating effect didn’t exist in other emotional dimensions. These results remained the same after accounting for the age and sex demographic variables.

### PA Is Associated With Decreased SWKN in the FPCN

**FIGURE 4:**
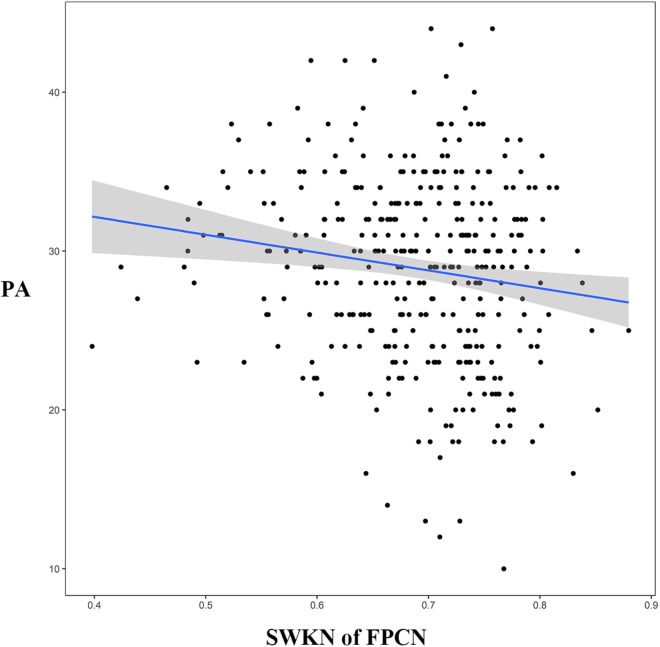
The relationship between SWKN of the FPCN and PA (subscale of the PANAS). SWKN = segregation within key nodes, FPCN = frontoparietal control network, PA = positive affect, PANAS = Positive Affect and Negative Affect Scale.

Two-tailed independent-samples *t*-tests suggested that there were significant differences in the SWKN of the FPCN between the HMW group and the LMW group (*t* = -3.55, *p* < 0.05, FDR corrected; see Table 1 for details). The following correlation analysis revealed that the SWKN of the FPCN was significantly negatively correlated with PA (*r* = -0.15, *p* < 0.05, FDR corrected) but not with NE (Figure 4).

**Table 1:** Means and standard deviations for the SWKN of different networks.

	HMW group mean(s.d.)	LMW group mean(s.d.)	Difference between HMW group and LMW group
SWKN of DMN	0.27(0.10)	0.29(0.11)	*t* = -1.73, *p* = 0.09
SWKN of COTC	0.14(0.32)	0.17(0.24)	*t* = -1.14, *p* = 0.25
SWKN of FPCN	0.68(0.08)	0.70(0.07)	*t* = -3.30, *p* < 0.05 (FDR corrected)
SWKN of DAN	0.06(1.20)	-0.08(1.23)	*t* = 1.13, *p* = 0.26
SWKN of VAN	0.52(0.27)	0.54(0.19)	*t* = -0.57, *p* = 0.57
SWKN of SN	0.39(0.18)	0.39(0.16)	*t* = 0.03, *p* = 0.98


*HMW = high mind wandering, LMW = low mind wandering, SWKN = segregation within key nodes, DMN = default mode network, COTC = cingulo-opercular task control network, FPCN = frontoparietal control network, DAN = dorsal attention network, VAN = ventral attention network, SN = salience network.*

## Discussion

As mind wandering occurs in many situations and makes up an important component of an individual’s waking hours, this study partly demonstrated the neural mechanisms of mind wandering and emotions. The current study aimed to explore the brain patterns of the relationships among mind wandering, thought control ability and emotions from the perspective of individual differences. There are three major results: first, we found that selecting CF with three different thresholds could successfully classify individuals with higher or lower levels of MW, TCA, PE and NE, meaning that adopting biomarkers related to mind wandering and thought control ability to define subtypes of students was efficient (Supplementary Materials); second, the finding that TCA moderated the relationship between MW and PA indicates that TCA is a crucial psychological trait in deciding the effect of MW on PA; and finally, the last results were the HMW group and the LMW group had different SWKN levels of the FPCN among six networks, PA was negatively correlated with the SWKN of the FPCN.

Connectivity biomarkers associated with TCA and MW could efficiently classify people with significantly different degrees of MW, TCA, PE and NE, and the results also proved that these four characteristics were highly correlated (Peterson et al., 2009; Killingsworth and Gilbert, 2010; Brewer et al., 2011; Smallwood and O’Connor, 2011; Stawarczyk et al., 2012; Gootjes and Rassin, 2014). The results also indicated that using resting-state connectivity biomarkers to define subtypes of emotion was efficient. Mind wandering and thought control ability have been discussed as phenomena accompanied by recruitment of executive control function (Levy and Anderson, 2008; Mcvay and Kane, 2009; Williams et al., 2010). Biomarkers selected by these two psychological characteristics were used to successfully classify people with different levels of positive or negative emotion, this result aligned with previous findings that emotion has an interaction with executive control (Noga and Avishai, 2012; Stollstorff et al., 2013).

Perceived thought control ability could predict cognitive control (Williams et al., 2010). A series of studies provided evidence that low levels of control often predict relatively high rates of mind-wandering (Mcvay and Kane, 2009; Unsworth and Mcmillan, 2013). In addition, the negative mind wandering content is associated with subsequent worse feelings (Poerio et al., 2013). TCA moderated the effects of MW on PA, indicating that individuals with low TCA scores experienced a bigger decrease in PA than participants with high TCA scores, one of the possible reasons is that participants who obtained higher scores on the TCAQ are happier because they inhibited their unpleasant thoughts during mind wandering. The other explanation is that high thought control ability reduced the tendency to engage in mind wandering, which resulted in more positive affect (Brewer et al., 2011). Future- and self-related mind wandering is related to improvements of mood (Ruby et al., 2013a), Engert et al. consolidated the conclusion that focuses on the future is linked to more positive aspects (Engert et al., 2014), individuals with higher thought control ability may have more mind wandering with content regarding future and self. The modulating effect exists only in the LMW group but not in the HMWgroup, considering the different FPCN of SWKN between two groups, we suppose TCA and FPCN are key variables in the relation between MW and PA. However, this phenomenon did not exist in the MW and NE relationship, meaning that TCA is an important factor in the relationship between MW and PA rather than in negative cognitive aspects.

The greater the PA, the lower the level of SWKN between the FPCN and other mind wandering or thought control ability regions, this result explained the brain mechanisms of MW, TCA and PA. The FPCN is a task-positive network consisting of many executive control regions (Vincent et al., 2008; Raichle, 2015). The SWKN of the FPCN represents values of within-network of FPCN correlations in relation to between-network correlations. This finding provides supporting evidence that the intention of mind wandering is linked to the FPCN (Golchert et al., 2017). It’s also consistent with the result that global connectivity involving not only within but also outside connections of a component in FPCN predicts cognitive control (Cole et al., 2012). The executive failure hypothesis of mind wandering is one of the widely known theories to shed light on the act of mind wandering (Mcvay and Kane, 2009, 2010, 2012). We explain this result as an executive failure of the FPCN in MW- and TCA-related regions that lead to higher levels of MW (Corbetta et al., 2008; Mittner et al., 2016), which resulted in a lower level of PA (Carciofo et al., 2017). Executive control is known to play an important role in the regulation of cognition (Duncan, 2010), positive emotion and cognitive control regions exhibit interplay (Stollstorff et al., 2013), and there exists a different interpretation that the FPCN may suppress the negative content of mind wandering so that individuals feel happier. Furthermore, content regulation hypothesis, which argues that the ability to regulate the content of mind-wandering to entail less upsetting topics is thought to depend on executive control (Smallwood and Andrewshanna, 2013; Smallwood and Schooler, 2015), is also an explanation for these results considering we didn’t put the content of mind-wandering variables into this study. We consider that a combination of the FPCN’s SWKN and thought control ability differences causes PA changes.

Our study used neuroimaging biomarkers to partly reveal how mind wandering affects mood, which is crucial for researchers to understand the mechanism of mind wandering and to do research on mind wandering in the future. However, Large-scale brain networks were employed to research mind wandering, as mind wandering is an activity requiring whole-brain involvement. As for data analysis, we didn’t use the traditional method of functional connection, the SWKN was used for exploring the relationships between networks of interest and whole brain networks. Recently, studies in mounting numbers have indicated the function of white matter in the brain network (Ji et al., 2017). There are also some works that have applied resting-state fMRI to investigate the function of white matter in brain disorders (Ji et al., 2018). These studies demonstrated that the functional connectivity of white matter may be a good indicator of behavior assessments. And we will focus on this indicator in future studies. Results may be facilitated in the future by considering whether the content of emotional mind-wandering can be classified by the orbito-frontal cortex (Tusche et al., 2014). The lack of a replicated dataset is a downside of this study, and we will compensate for that in future studies. Moreover, the TCA we defined is only based on TCAQ, and it can be strengthened in the future. The study relied on questionnaires of thought control, mind wandering, affect, etc,. It would be much stronger if behavioral or other measures were also included. There were 10 functional connections that have been shown to be crucial for classifying individuals, meaning that these connections are the key nodes, and should be studied in depth to determine what these brain features mean in terms of mind wandering. These important compositions were crucial when studying task-fMRI studies and other neuroimaging forms of studies on mind wandering in the future. Furthermore, the exposure theory hypothesizes that future well-being is a result of the expression of negative emotion (Frattaroli, 2006) - whether suppressing negative emotions contributes to good health needs further discussion.

## Conclusion

In conclusion, this research concluded that thought control ability moderated the relationships between mind wandering and emotions by reallocating resources of the executive control networks. MW- and TCA-related regions classified people with different degrees of emotions. The FPCN was the critical network that contributes to the relationship between MW and PA. Executive control was a common theoretical basis for mind wandering and thought control ability. Thought control ability moderated the relationship between mind wandering and PA, and the FPCN’s SWKN differences regarding these two psychological traits. The related ROIs across the whole brain reinforced the conclusion. Despite the limitations of the current results, the use of neuroimaging biomarkers to explore the relationships among mind wandering, thought control ability and emotions was unique. These results remind us to focus on executive control cognitive ability while researching mind wandering and emotions. These results provide us with insights into the neural basis of mind wandering and provide a viable method for increasing happiness by influencing the characteristics of mind wandering.

## Ethics Statement

This study was carried out in accordance with the recommendations of “Institutional Review Board of the Southwest University Brain Imaging Center” with written informed consent from all subjects. All subjects gave written informed consent in accordance with the Declaration of Helsinki. The protocol was approved by the “Institutional Review Board of the Southwest University Brain Imaging Center”.

## Author Contributions

JQ provided technical support for conducting the experiments. HH, QC, and JQ put forward the academic problem. HH contributed to the recruitment of subjects, data acquisition, and completing most of the manuscript. DW and LS revised the manuscript.

## Conflict of Interest Statement

The authors declare that the research was conducted in the absence of any commercial or financial relationships that could be construed as a potential conflict of interest.

## Footnotes

1 http://restfmri.net

2 https://www.fil.ion.ucl.ac.uk/spm/

3 https://cran.r-project.org/web/packages/jtools/vignettes/interactions.html

4 http://cn.mathworks.com/help/stats/evalclusters.html

5 https://cran.r-project.org/web/packages/MASS
