# Supplementary material for: Thought Control Ability Moderates the Effect of Mind Wandering on Positive Affect via the Frontoparietal Control Network
Source: Front Psychol. 2019 Jan 25;9:2791. doi: 10.3389/fpsyg.2018.02791 (PMC6357679; doi:10.3389/fpsyg.2018.02791)
Supplement: Supplementary file 1 [file Table_1.DOCX]

[**Supplementary**](http://cn.bing.com/dict/clientsearch?mkt=zh-CN&setLang=zh&form=BDVEHC&ClientVer=BDDTV3.5.0.4311&q=%E8%BE%85%E5%8A%A9%E6%9D%90%E6%96%99) [**material**](http://cn.bing.com/dict/clientsearch?mkt=zh-CN&setLang=zh&form=BDVEHC&ClientVer=BDDTV3.5.0.4311&q=%E8%BE%85%E5%8A%A9%E6%9D%90%E6%96%99)**s**

**Supplementary Table 1.** Descriptive statistics for all measures.

| Measure | Mean(SD) | Range | Skewness | Kurtosis |
| --- | --- | --- | --- | --- |
| DDFS | 17.55(8.58) | 0-48 | 0.69 | 0.67 |
| MWFS | 54.88(16.49) | 21-105 | 0.48 | -0.18 |
| PA | 28.95(5.83) | 10-50 | -0.23 | 0.013 |
| NA | 19.82(5.58) | 10-50 | 1.01 | 1.64 |
| BAI | 5.41(5.10) | 21-84 | 1.93 | 5.84 |
| BDI | 8.14(6.28) | 0-21 | 1.10 | 0.92 |
| UHIS | 78.34(11.43) | 28-140 | -0.44 | 1.39 |
| OHI | 118.07(15.07) | 29-174 | -0.05 | -0.08 |
| CES-D | 14.94(8.25) | 0-60 | 0.57 | -0.14 |
| TCAQ | 76.08(12.55) | 25-125 | -0.01 | 0.42 |

Note. DDFS = daydreaming frequency, MWFS = mind wandering frequency scale, PA = positive affect, NA = negative affect, BAI = beck anxiety inventory, BDI = beck depression inventory, UHIS = urban happiness index scale, OHI = oxford happiness inventory, CES-D = center for epidemiological studies-depression scale, TCAQ = thought control ability questionnaire.


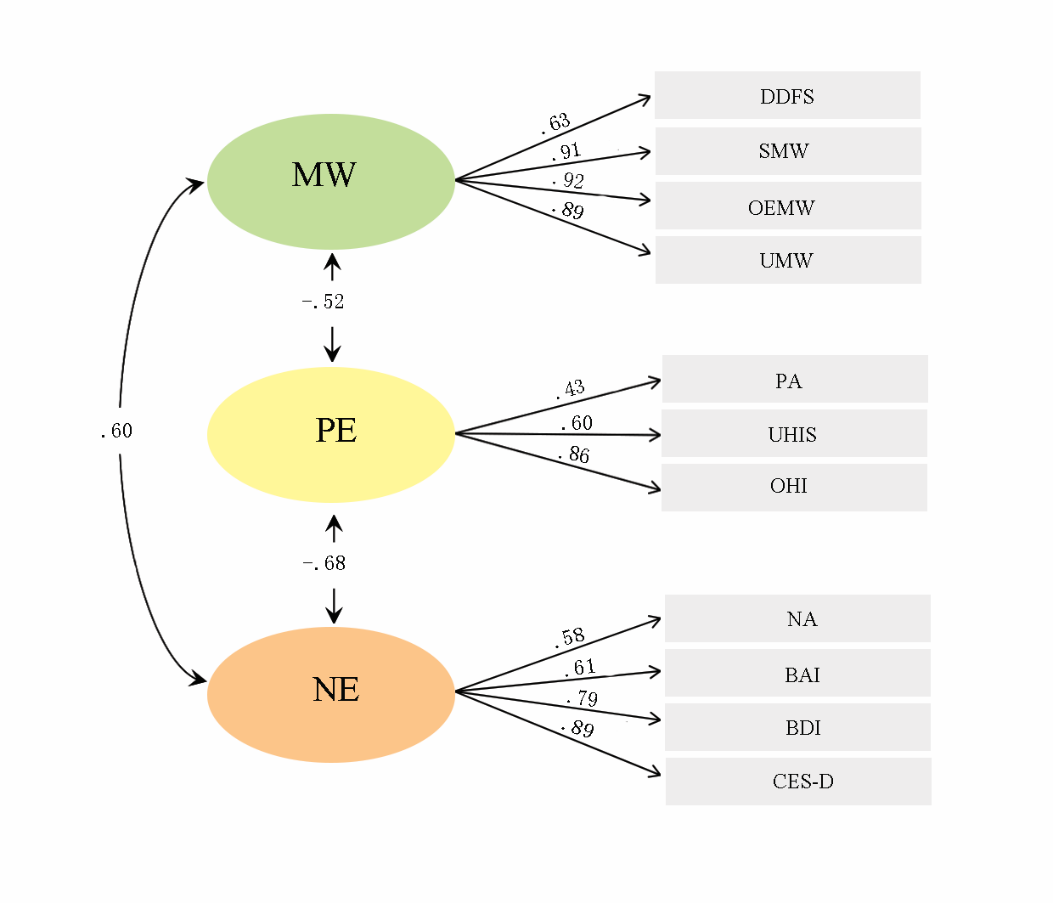


**Supplementary Figure 1.** Standardized factor loadings for the confirmatory analysis for all measures of MW, PE, and NE. MW = mind wandering, PE = positive emotion, NE = negative emotion, DDFS = daydreaming feaquency scale, SMW = spontoneous mind wandering (one dimension of mind wandering frequency scale), OEMW = overall evaluation of mind wandering (one dimension of mind wandering frequency scale), UMW = uncontrol mind wandering (one dimension of mind wandering frequency scale), PA = positive affect (one dimension of positive affect and negative affect scale), UHIS = urban happiness index scale, OHI = oxford happiness inventory, NA = negative affect (one dimension of positive affect and negative affect scale), BAI = beck anxiety inventory, BDI = beck depression inventory, CES-D = center for epidemiological studies-depression scale.

We constructed T maps with 0.005 and 0.01 thresholds for the P values between the functional connectivity and the behavioral index. Specifically, the CF included 216 ROIs with a 0.005 threshold and 242 ROIs with a 0.01 threshold. In addition, the following maps are T maps of positive functional connectivity and negative functional connectivity.


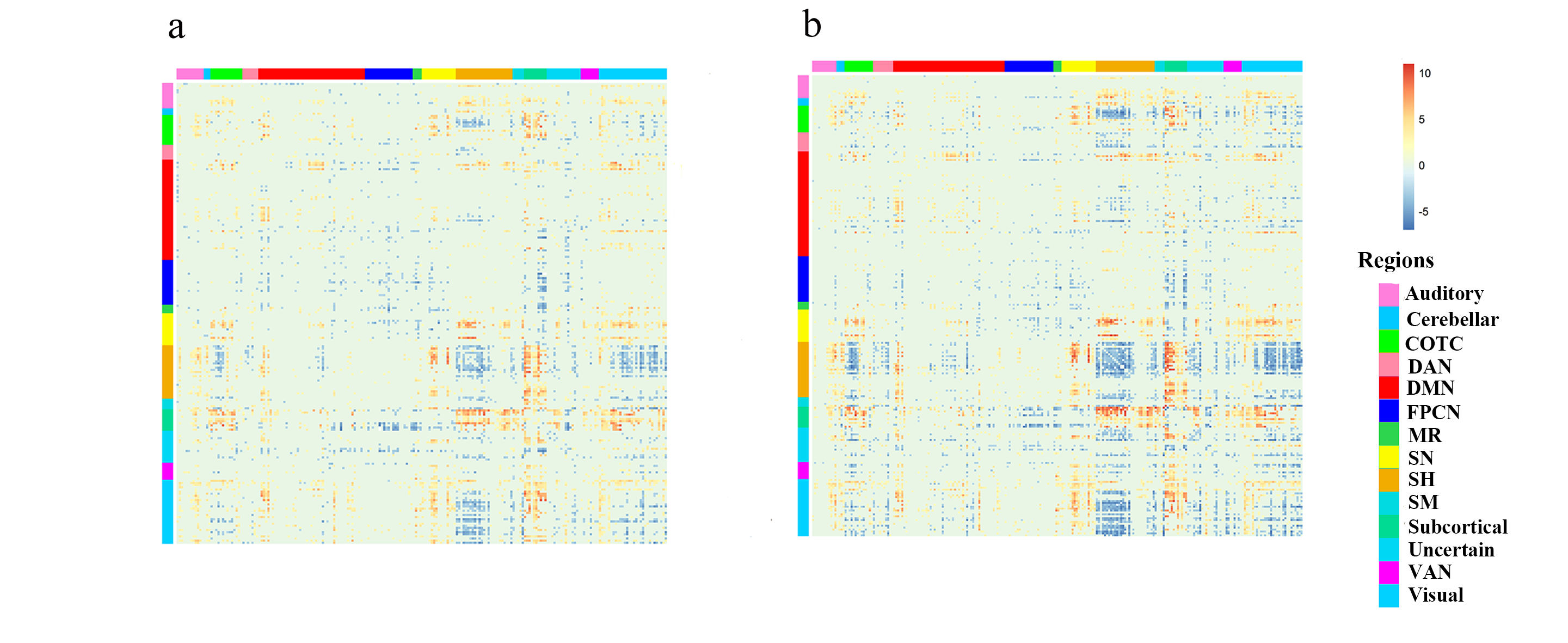


**Supplementary Figure 2.** T-test matrix of the two groups regarding positive functional connectivity with two thresholds. (a) The 216 × 216 t-test matrix of the two groups in terms of positive functional connectivity with a 0.005 threshold (FDR corrected). (b) The 242 × 242 t-test matrix of the two groups in regard to positive functional connectivity with a 0.01 threshold (FDR corrected). Each colored square represents the significant t value of one functional connectivity between two groups. The X-axis and Y-axis represents 95 nodes respectively, warm colors in the matrix represent increased functional connectivity and cool colors decreased functional connectivity in HMW group as compared to LMW group. Network affiliation abbreviations: Auditory = Auditory network, Cerebellar = cerebellar network, COTC = cingulo-opercular task control network, DAN = dorsal attention network, DMN = default mode network, FPCN = frontoparietal control network, MR = memory retrieval network, SN = salience network, SH = somatomotor hand network, SM = somatomotor mouth network, VAN = ventral attention network. Subcortical = subcortical network, Uncertain = uncertain network, and Visual = visual network.

The selected CF within the spontaneously related networks were 10 functional connectivities (Supplementary fig. 3), and this subset could acceptably predict the classification results of the whole-brain CF, with an accuracy rate of 0.68. In addition, the Calinski-Harabasz criterion value showed that the optimal number of the group was also two. Two-tailed [independent](http://cn.bing.com/dict/clientsearch?mkt=zh-CN&setLang=zh&form=BDVEHC&ClientVer=BDDTV3.5.0.4311&q=%E5%8F%8C%E6%A0%B7%E6%9C%ACt%E6%A3%80%E9%AA%8C)-samples t-[test](http://cn.bing.com/dict/clientsearch?mkt=zh-CN&setLang=zh&form=BDVEHC&ClientVer=BDDTV3.5.0.4311&q=%E5%8F%8C%E6%A0%B7%E6%9C%ACt%E6%A3%80%E9%AA%8C)s suggested that these 10 CF could successfully divide people among MW, TCA, and NE but not PE. (Supplementary fig. 4)


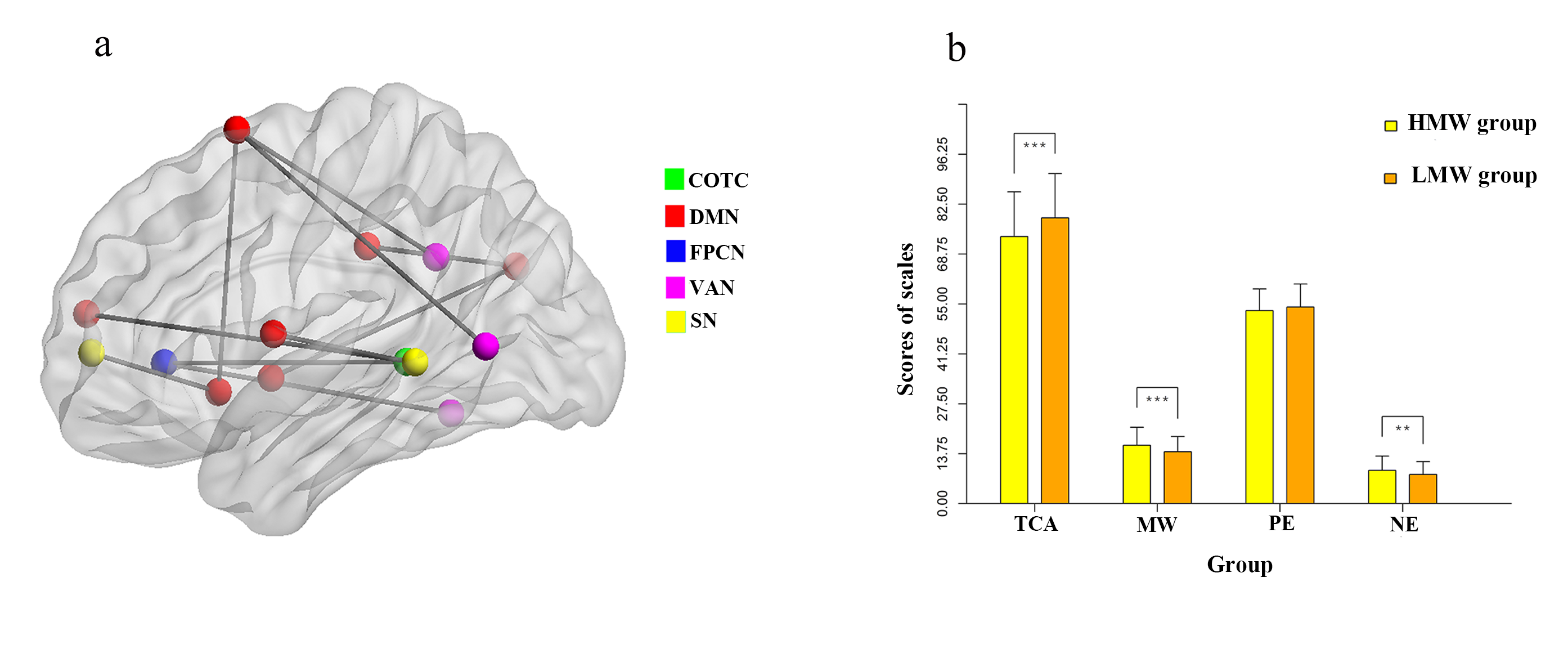


**Supplementary Figure 3.** The 10 functional connectivities of the five networks of interest could acceptably classify individuals. (a) The neuroanatomical distribution of the CF of the 6 networks. (b) The t-test histogram of each componet (as indexed by the CFA solution) between the two groups divided by using the CF belonging to the 6 networks of interest that were considered higher vs. lower in MW. Network affiliation abbreviations: COTC = cingulo-opercular task control network, DAN =dorsal attention network, DMN = default mode network, FPCN = frontoparietal control network, SN = salience network, VAN = ventral attention network, CF = connectivity features, HMW= high mind-wandering, LMW =low mind-wandering. *** p ≤ 0.001, ** p ≤ 0.01.


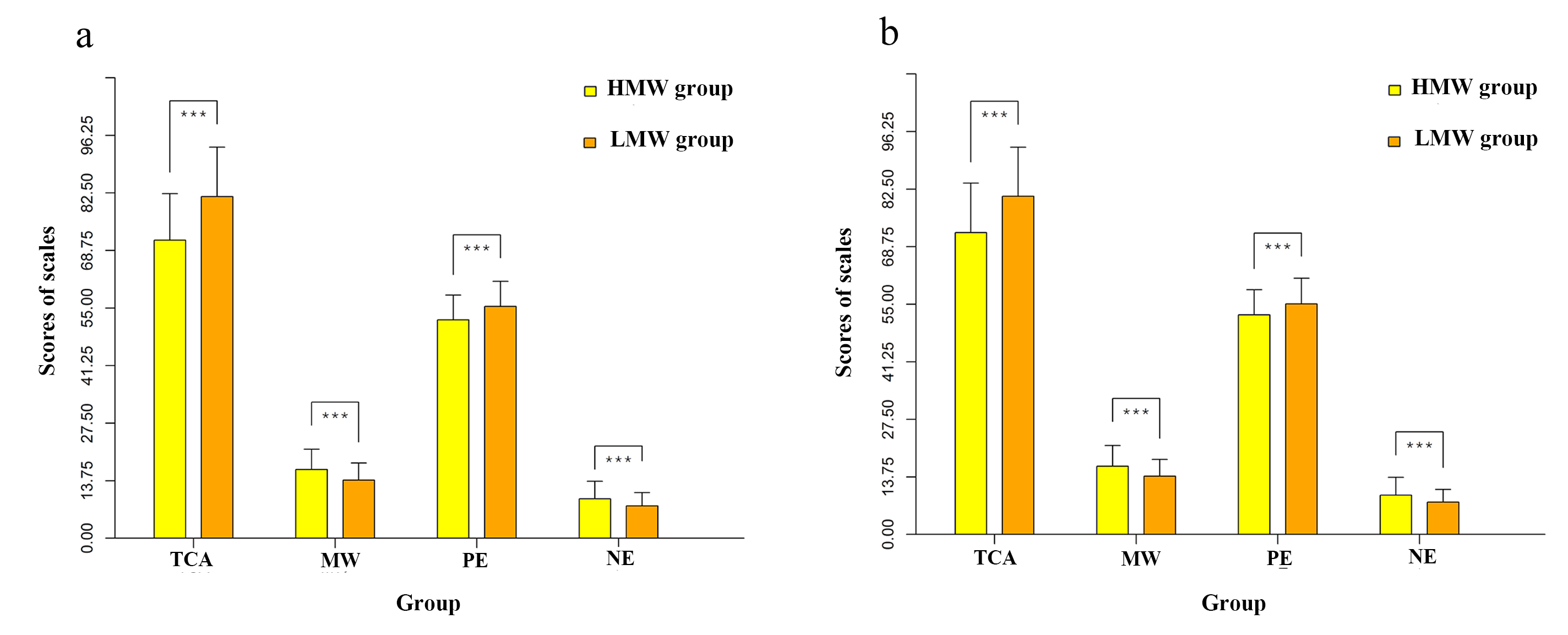


**Supplementary Figure 4.** The different groups of participants divided by CF at significance thresholds of 0.005 (a) and 0.01 (b) into the four-dimensional scores (as indexed by the CFA solution) that were considered higher vs. lower in MW. MW = mind wandering, TCA = thought control ability, PE = positive emotion, NE = negative emotion HMW= high mind-wandering, LMW =low mind-wandering. *** p ≤ 0.001.


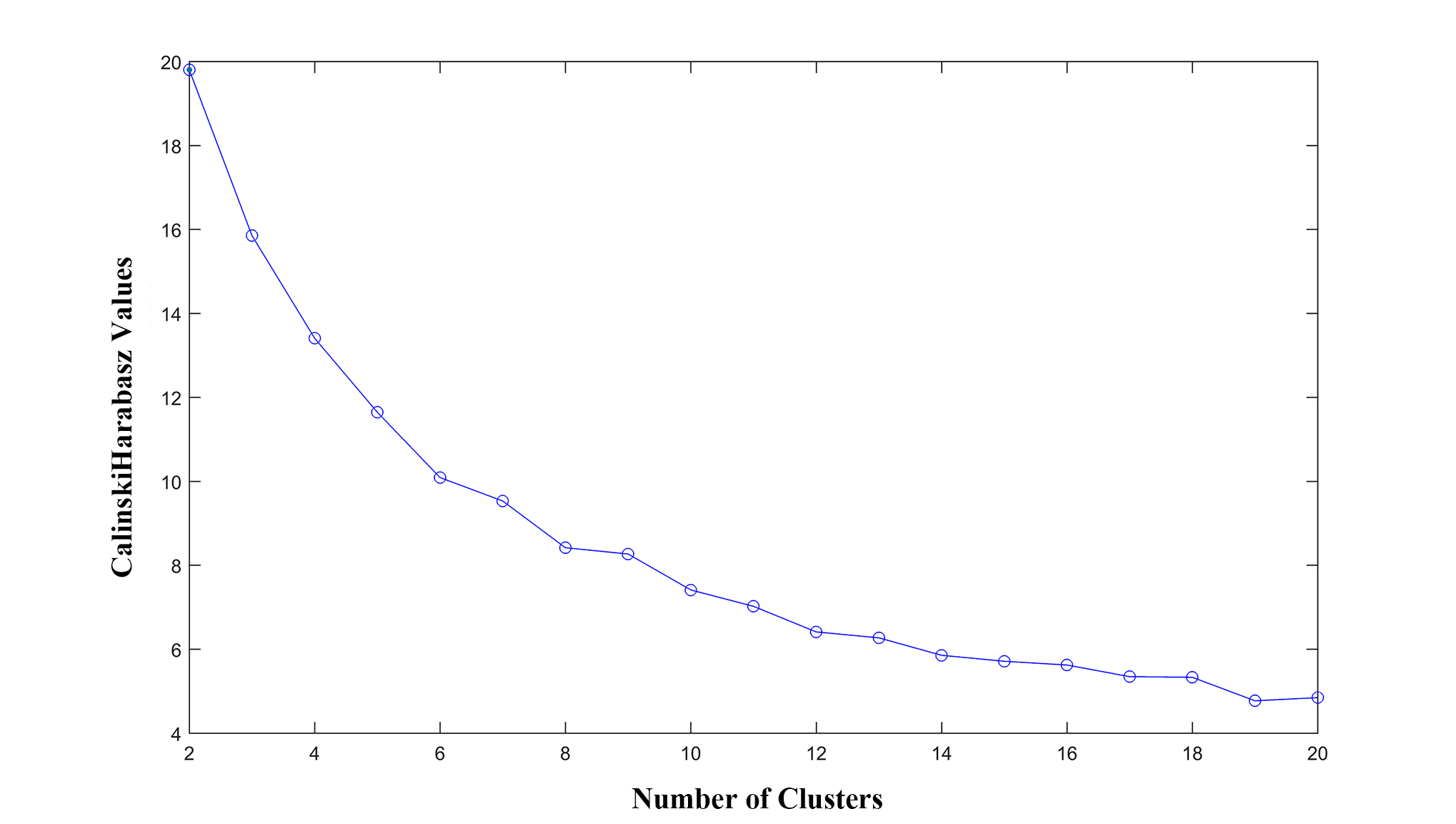


**Supplementary Figure 5.** The line graph of calinskiHarabasz values with cluster numbers betlinween 1-20.
